# Biochemical Mechanisms, Cross-resistance and Stability of Resistance to Metaflumizone in *Plutella xylostella*

**DOI:** 10.3390/insects11050311

**Published:** 2020-05-15

**Authors:** Jun Shen, Zhao Li, Dongyang Li, Rumeng Wang, Shuzhen Zhang, Hong You, Jianhong Li

**Affiliations:** 1 College of Horticulture, Xinyang Agriculture and Forestry University, Xinyang 464000, China; shenjun996@163.com; 2 Hubei Insect Resources Utilization and Sustainable Pest Management Key Laboratory, College of Plant Science and Technology, Huazhong Agricultural University, Wuhan 430070, China; Lucky_edmund@163.com (Z.L.); hzaulidongyang@163.com (D.L.); ru_meng_wang@163.com (R.W.); shuzhen7@126.com (S.Z.); youhong@mail.hzau.edu.cn (H.Y.)

**Keywords:** detoxification enzymes, cross-resistance, metaflumizone, *Plutella xylostella*, resistance

## Abstract

The diamondback moth, *Plutella xylostella* (L.) is an important pest of cruciferous crops worldwide. It has developed resistance to many conventional and novel insecticide classes. Metaflumizone belongs to the new chemical class of semicarbazone insecticides. To delay the development of metaflumizone resistance in *P. xylostella* and to guide insecticide use in the field, the biochemical mechanisms, cross-resistance spectrum, and stability of resistance to metaflumizone were studied in a laboratory-selected resistant strain (metaflu-SEL). Synergism tests with the carboxylesterase inhibitor triphenyl phosphate (TPP), the glutathione S-transferase depletor diethyl maleate (DEM), and the P450 inhibitor piperonyl butoxide(PBO) had no obvious effect on metaflumizone in the metaflu-SEL strain and the susceptible strain (SS) of *P. xylostella*, with synergism ratios that ranged from 1.02 to 1.86. Biochemical studies revealed that the cytochrome P450-dependent monooxygenase increased only 1.13-fold in the metaflu-SEL strain compared with the UNSEL stain; meanwhile, carboxylesterase and glutathione *S*-transferase activity showed no difference. These results suggest that these detoxification enzymes may be not actively involved in metaflumizone resistance. Furthermore, the metaflu-SEL population showed a moderate level of cross-resistance to indoxacarb (11.63-fold), but only very low cross-resistance to spinosad (1.75-fold), spinetoram (3.52-fold), abamectin (2.81-fold), beta-cypermethrin (0.71-fold), diafenthiuron (0.79-fold), chlorantraniliprole (2.16-fold), BT (WG-001) (3.34-fold), chlorfenapyr (0.49-fold), and chlorfluazuron (0.97-fold). Moreover, metaflumizone resistance decreased from 1087.85- to 1.23-fold in the metaflu-SEL strain after 12 generations without exposure to metaflumizone. These results are useful for formulating insecticide resistance management strategies to control *P. xylostella* and to delay the development of metaflumizone resistance in the field.

## 1. Introduction

The diamondback moth (DBM), *Plutella xylostella* (Lepidoptera: Plutellidae), is one of the most destructive cosmopolitan pests of cruciferous crops. Its annual management costs and associated crop losses are estimated to be $4–5 billion worldwide and approximately $0.77 billion in China [1,2]. Because of the irrational use of chemical insecticides, *P. xylostella* has developed different levels of resistance to various insecticides [3,4]. Based on the latest data from APRD, *P. xylostella* has developed resistance to 97 compounds, and *P. xylostella* was ranked first of the top 20 most resistant species [5].

As a member of the new chemical class of semicarbazone insecticides, Metaflumizone blocks the sodium channels of insects by binding selectively to the slow-inactivated state of the channels, causing flaccid paralysis and the eventual death of the target insects [6,7,8]. Metaflumizone has been used to effectively control a wide range of pests [9]. As an Environmental Protection Agency (EPA) reduced-risk candidate, metaflumizone was registered by BASF Chemical Co. in China in 2009 to control *P. xylostella* and *Spodoptera exigua* (Lepidoptera: Noctuidae) on *Brassica* vegetables [10]. Field populations of *S. exigua* have developed high level of resistance to metaflumizone [11]. In contrast, Khakame reported that *P. xylostella* collected from 14 geographical locations in China showed 1- to 3-fold resistance to metaflumizone [12]. However, field populations of *P. xylostella* have developed high levels of resistance (250- to 870-fold) to indoxacarb and medium levels of cross-resistance (10- to 70-fold) to metaflumizone compared with the susceptible strain [13]. These reports indicate that *P. xylostella* has the potential to develop high levels of resistance to metaflumizone in the field.

To effectively use metaflumizone to manage *P. xylostella* and to develop an effective strategy in integrated pest management (IPM) programs that will delay the development of resistance to metaflumizone in the field, it is necessary to study the biochemical mechanisms, the cross-resistance, and the stability of resistance in laboratory-selected metaflumizone resistant strain. Therefore, in this study, enzymatic and synergism assays were performed to elucidate the biochemical mechanisms of metaflumizone resistance in the *P. xylostella*. Cross-resistance to indoxacarb, abamectin, beta-cypermethrin, chlorantraniliprole, and other insecticides was determined in a laboratory-selected strain of *P. xylostella* with high levels of resistance to metaflumizone. Additionally, the stability of resistance to metaflumizone was investigated in the absence of metaflumizone selection pressure. 

## 2. Materials and Methods

### 2.1. Insects

The susceptible strain (SS) and the resistant strain (metaflu-SEL) have been described (published previously) [14]. The population of metaflu-SEL larvae were used to investigate cross-resistance, synergistic effects, stability of resistance, and enzyme activity (according to the number of larvae). The resistance decaying strain (UNSEL), a revertant strain, was derived from a substrain of metaflu-SEL that had not been exposed to metaflumizone or any other insecticide for 12 consecutive generations. The larvae were reared on vermiculite-grown radish (*Raphanus sativus* L.) seedlings, and the adults were provided with a 10% honey/water solution in the laboratory under controlled conditions of 25 ± 1 °C, 65 ± 5% RH and a 16:8 h L:D photoperiod in a separate greenhouse.

### 2.2. Chemicals

Metaflumizone (240 g/L SC) was obtained from the BASF Chemical Co., Ltd. (Shanghai, China). Indoxacarb (95%), abamectin (95%), beta-cypermethrin (96.1%), chlorfluazuron (95%), chlorfenapyr (95%), diafenthiuron (98%), and chlorantraniliprole (95%) were purchased from Hubei Kangbaotai Fine-Chemicals Co., Ltd. (Wuhan, China). Spinetoram (60 g/L SC) and spinosad (25 g/L SC) were purchased from the Dow AgroSciences Co., Ltd. (Shanghai, China). BT WG-001 (16000 IU/mg) was supplied by the Hubei Biopesticide Engineering Research Center. The following were obtained from the Sigma Chemical Co., Ltd. (St. Louis, MO, USA): triphenyl phosphate (TPP, reagent grade); diethyl maleate (DEM, reagent grade); piperonyl butoxide (PBO, reagent grade); 1-chloro-2,4-dinitrobenzene (CDNB); reduced glutathione (GSH); fast blue B salt; sodium-dodecyl sulphate (SDS); dithiothreitol (DTT); eserine; phenylmethanesulfonyl fluoride (PMSF). Alpha-naphthol acetate (α-NA) and ethylene diamine tetra-acetic acid (EDTA) were purchased from the Sinopharm Chemical Reagent Co., Ltd. (Shanghai, China). Reduced nicotinamide adenine dinucleotide phosphate (NADPH) was obtained from Roche Molecular Systems, Inc. (San Francisco, CA, USA). The protein-assay dye reagent was provided by Bio-Rad Laboratories, Inc. (Shanghai, China).

### 2.3. Bioassay

The leaf-dipping bioassay was used to determine the susceptibility of the third instar larvae of *P. xylostella* to insecticides (According to “Guideline for insecticide resistance monitoring of *Plutella xylostella* (L.) on cruciferous vegetables) [15,16]. The insecticide was diluted to generate five to seven serial dilutions with water containing 0.1% Triton X-100 to facilitate a uniform leaf disc coverage with the active ingredient. Cabbage leaf discs (7.0 cm diameter) were cut and dipped in an insecticide solution for 10 s. Control discs were treated with a 0.1% Triton X-100 solution in water. The leaf discs were dried at room temperature for 2 h. Each treated leaf disc with 15 third instar larvae was placed in a separate plastic Petri dish and kept at 25 ± 1 °C with an RH of 60 ± 5% and a 16:8 h L:D photoperiod. For each insecticide concentration, three replicates of larvae were used. The mortality was assessed after 48 h expose to indoxacarb, spinosad, spinetoram, abamectin, beta-cypermethrin, chlorfenapyr, diafenthiuron, and chlorantraniliprole, as well as 72 h expose to BT (WG-001), chlorfluazuron, and metaflumizone. Larvae were considered dead if they could not move when touched with a fine brush. The control mortality was less than 5% in all bioassays. 

To analyze the synergistic effects of enzyme inhibitors with metaflumizone, a total of 200 mg/L of PBO, DEM, and TPP which did not result in larval mortality were added to separate aliquots of each dilution. Bioassays were conducted again for the metaflu-SEL and SS populations at the 27th generation (G_27_, RR = 1338.99-fold) with and without synergists. To assess the degree of synergism, the synergistic ratio (SR) was calculated by dividing the LC_50_ value of metaflumizone alone by the LC_50_ value of metaflumizone with the synergist treatments.

### 2.4. Enzyme Activity Assays

Carboxylesterase (CarE) activity was determined using the method of Asperen (1962) with modification [17]. Ten fourth instar larvae of *P. xylostella* were homogenized in 1 mL of ice sodium phosphate buffer (0.04 M, pH 7.0) and centrifuged at 4 °C, 11,000 rpm for 15 min. The homogenized supernatant was then carefully transported to a new Eppendorf tube and was used as the enzyme source. Total of 1.8 mL of substrate solution (containing 3 × 10^−4^ M α-NA and 3 × 10^−4^ eserine), 450 μL of sodium phosphate buffer (0.04 M, pH 7.0), and 50 μL of diluted supernatant (diluted 10-fold) were added to the Eppendorf tube and incubated at 30 °C for 15 min. The reaction was stopped with the addition of 900 μL of dye reagent (1% fast blue B salt: 5% SDS = 2:5 V/V). The optical density (OD) was recorded at 600 nm using an ultraviolet spectrophotometer (SHIMADZU UV-1800). 

Glutathione *S*-transferase activity was determined as previously described by Habig [18]. The enzyme solution was prepared with ten fourth instar of *P. xylostella* frozen larvae (−80 °C) homogenized in sodium phosphate buffer (0.1 M, pH 6.5) and centrifuged at 1000 r/min for 10 min at 4 °C. The supernatant was collected as the crude enzyme solution. For each reaction, 810 μL of sodium phosphate buffer (0.1 M, pH 6.5), 30 μL of 30 mM 1-chloro-2,4-dinitrobenzene (CDNB), 50 µL of enzyme solution, 30 μL of 30 mM GSH were mixed. The absorbance was recorded using a NP80 NanoPhotometer (IMPLEN, Munich, Germany) at 340 nm for 2 min. The crude enzyme was diluted 40 times for the protein concentration determination. 

The 7-ethoxycoumarin-O-deethylase (7-ECOD) activity of P450 was determined as described previously [19] with modification. Ten fourth instar larvae were homogenized in 1.0 mL of sodium phosphate buffer (0.1 M sodium phosphate buffer, pH 7.5, containing 1 mM EDTA, 1 mM PMSF 1 mM DTT, and 10% glycerol) and then centrifuged at 4 °C 14,000 rpm for 15 min. The supernatant was added to a new Eppendorf tube and centrifuged at 4 °C 14,000 rpm for 30 min again. The supernatant was then used as the enzyme source. For the reaction, 685 μL of Tris-HCl buffer (0.1 M, pH 7.8), 20 μL of aqueous NADPH (10 mM) and 25 μL of 7-ECOD (2 mM, dissolved with absolute ethanol), and 250 μL of crude homogenate were mixed. After incubation at 30 °C for 15 min, 300 μL of 15% trichloroacetic acid was added to terminate the reaction. Then, the mixture was centrifuged, and 800 μL of the supernatant was extracted. The pH of the resulting extract was adjusted to approximately 10 by adding 400 μL of 1.6 mM glycine-NaOH buffer (pH 10.5). The fluorescent intensity was measured using a Spark 10 M Multimode Microplate Reader (Tecan, Männedorf, Switzerland) with an excitation wavelength of 358 nm and an emission wavelength of 465 nm. The P450 activity was calculated from a 7-hydroxycoumarin standard curve.

### 2.5. Cross-Resistance 

To determine the susceptibilities of the UNSEL and metaflu-SEL strains to other insecticides (including indoxacarb, spinosad, spinetoram, abamectin, beta-cypermethrin, diafenthiuron, chlorantraniliprole, BT (WG-001), chlorfenapyr, chlorfluazuron), the metaflu-SEL population was treated with the insecticides for up to G_22_ (RR = 953.98-fold) or G_23_ (RR = 942.78-fold) and the data were compared with the susceptible strain. Cross-resistance was determined by calculating the cross-resistance ratio (CR). The CR value was estimated as: CR = LC_50_ of metaflu-SEL strain / LC_50_ of UNSEL strain.

### 2.6. Stability of Resistance

To investigate the stability of resistance to metaflumizone, the UNSEL strain was reared for 12 generations (G_28_-G_40_) without insecticide exposure. The reduction in the resistance (DR) to insecticides of the UNSEL strain was estimated by calculating the *R* value using the following formula [20]:

$$R=(\mathrm{Log}\mathrm{final}{\mathrm{LC}}_{50}-\mathrm{Log}\mathrm{initial}{\mathrm{LC}}_{50})/n$$

where *n* is the number of generations without selection. Negative values of *R* reflect decreases in the LC_50_. The inverse of *R* is the number of generations required for a ten-fold change in the LC_50_.

### 2.7. Data Analyses

Bioassay data, including the LC_50_ values and the 95% confidence limits (CL), were calculated using probit regressions in the Probit-MS computer program [21]. Enzyme data were analyzed with Tukey’s test with a significance level of *p* < 0.05 using the IBM SPSS Statistical [22]. 

## 3. Results

### 3.1. Synergism of PBO, DEF, and DEM with Metaflumizone

The synergistic effects of PBO, DEF, and DEM on metaflumizone were tested for the metaflu-SEL strain and the SS of *P. xylostella* (Table 1). The inhibitors (PBO, TPP and DEM) had no obvious synergistic effects on metaflumizone in the metaflu-SEL strain of *P. xylostella*, with synergism ratios of 1.24-, 1.86-, and 1.42-fold, respectively. Similarly, no obvious synergistic effects of the inhibitors were also found in the SS. These results suggested that the effect of metaflumizone on the metaflu-SEL strain and the SS was not enhanced by PBO, TPP, or DEM. 

### 3.2. Activity of the Detoxification Enzymes in Susceptible and Metaflu-SEL Strains of P. xylostella

**Figure 1:**
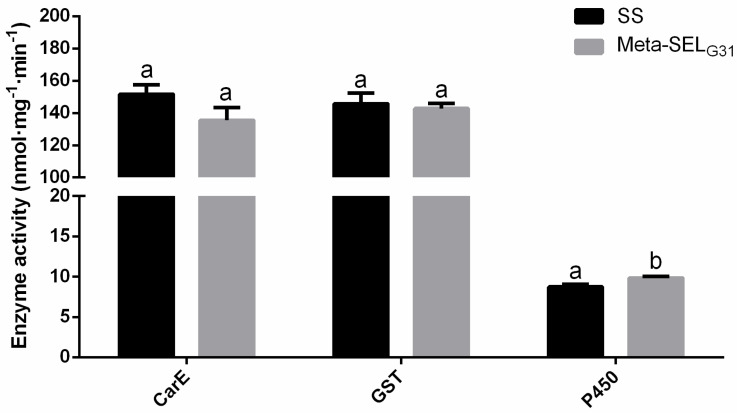
Activity of detoxification enzymes in the Meta-SELG31 and susceptible (SS) populations of *P. xylostella*. Error bars represent the standard error of the mean. Different small letter indicates significant difference at 0.05 level.

To determine the role of detoxification enzymes in metaflumizone resistance in the *P. xylostella*, enzyme assays were performed to measure the levels of CarE, GST, and P450 (Figure 1). No significant differences in the activities of CarE and GST were found between the susceptible and metaflu-SEL strains. The activity of the P450 increased only 1.13-fold in the metaflu-SEL strain compared with the SS, which suggests that P450 had a very limited effect on metaflumizone resistance in *P. xylostella*. 

### 3.3. Cross-Resistance of Metaflumizone to Different Conventional and New Chemical Insecticides

The metaflu-SEL strain and the SS were tested for cross resistance to other insecticides, including indoxacarb, spinosad, spinetoram, abamectin, beta-cypermethrin, chlorfenapyr, diafenthiuron, chlorantraniliprole, BT (WG-001), and chlorfluazuron (Table 2). The metaflu-SEL strain showed medium cross-resistance to indoxacarb (RR = 11.63-fold); however, there was no sign of cross-resistance to the other tested insecticides, including spinosad (1.75-fold), spinetoram (3.52-fold), abamectin (2.81-fold), chlorantraniliprole (2.16-fold) and BT (WG-001) (3.34-fold), beta- cypermethrin (0.71-fold), chlorfenapyr (0.49-fold), diafenthiuron (0.79-fold), and chlorfluazuron (0.97-fold).

### 3.4. Stability of Resistance

Metaflu-SEL populations of *P. xylostella* were reared for 12 generations from G_28_ to G_40_ without any insecticide exposure to determine whether the resistance to metaflumizone was stable. The results showed that metaflumizone resistance in the UNSEL strain was unstable after 12 generations without exposure to metaflumizone (Table 3). There was a significant decline in the LC_50_ values of metaflumizone tested after 12 generations (the 95% CI did not overlap). The rates of decrease in resistance to metaflumizone in the UNSEL populations (G_31_, G_32_, G_34_, G_35_, G_36_, G_39_, G_40_) were −0.13, −0.08, −0.11, −0.16, −0.21, −0.27, and −0.22, respectively. 

## 4. Discussion

The increased activity of detoxification enzymes such as CarE, GST, and P450 has been one of the most important factors of insect resistance [23]. Shono showed that P450 was one of the mechanisms of indoxacarb resistance in an indoxacarb resistant strain (RR > 118-fold) of *Musca domestica* (Diptera: Muscidae) in New York [24]. Indoxacarb resistance was also associated with P450 in *Spodoptera litura* [25], *Choristoneura rosaceana* [26], and *Helicoverpa armigera* [27]. Sayyed and Wright (2006) found that esterase was related to indoxacarb resistance in a field population of *P. xylostella* in Malaysia [28]. Esterases may also play a primary role in conferring metabolic resistance to metaflumizone in field populations of *S. exigua* in China and populations of *Tuta absoluta* in Ankara [29,30]. Nehare confirmed that GST was associated with indoxacarb resistance in *P. xylostella* [31]. Gao stated that esterase and glutathione *S*-transferases had a stronger detoxification effect in the RR-indox strain [32]. Wang claimed that both oxidases and esterases may be involved in the indoxacarb resistance in the SY14 strain [33]. Similarly, indoxacarb resistance was found to be associated with both mono-oxygenases and esterases activity in *Phenacoccus solenopsis* in Pakistan [34]. However, in the present study, enzymatic and synergism assays revealed that CarE, GST, and P450 were not actively involved in metaflumizone resistance in *P. xylostella*, suggesting that target-site resistance may be a major mechanism of metaflumizone resistance in metaflu-SEL population of *P. xylostella* [33]. These results indicate that different resistance mechanisms against sodium channel blocker insecticides (SCBIs) could exist in different insect species.

Resistance against one particular insecticide may also cause resistance to other insecticides because of a common or single mechanism of resistance [35]. Metaflumizone is a novel sodium channel blocker insecticide that shares a common mode of action with indoxacarb [7]. Therefore, selection of indoxacarb may confer cross-resistance to metaflumizone. For instance, the BY12 population of *P. xylostella* developed a 750- and 70-fold resistance to indoxacarb and metaflumizone, respectively [12]. Similarly, Su and Sun reported that because of the excessive and frequent use of metaflumizone, the HZ11 colony of *S. exigua* developed a 942- and 15.7-fold resistance to metaflumizone and indoxacarb, respectively [29]. Pan found that a field population of *S. litura* had a high level of resistance to indoxacarb (RR = 73.2-fold), and a low level of resistance to metaflumizone (6.95-fold) [36]. Moreover, medium cross-resistance to indoxcarb (22-fold) and metaflumizone (17-fold) was detected in the GR-IndR population of *T. absoluta* [37]. In the present study, very low cross resistance was observed between metaflumizone and other compounds such as spinosad, spinetoram, abamectin, chlorantraniliprole, BT (WG-001), beta-cypermethrin, chlorfenapyr, diafenthiuron, and chlorfluazuron, while medium cross-resistance was observed between metaflumizone and indoxacarb in the metaflu-SEL population of *P. xylostella*. These results suggest that the rotation of metaflumizone with other different modes of action may reduce the selection pressure caused by using a single product, delay the development of resistance, and ultimately prolong the efficacy of metaflumizone. 

It is critical to study resistance stability for the development of successful resistance management strategies [38,39]. However, information about the stability of resistance to SCBIs is limited. The reversal of indoxacarb resistance has been previously reported in *Spodoptera litura* [23] and *P. solenopsis* [40]. In the present study, the mateflumizone resistance level significantly declined from 1087.85- to 1.23-fold after 12 generations in the absence of selection, suggesting that metaflumizone resistance in *P. xylostella* was also unstable. 

## 5. Conclusions

The present study demonstrated that CarE, GST, and P450 were not actively involved in the metaflumizone resistance. Additionally, a medium cross-resistance to indoxacarb in the metaflu-SEL population and an unstable metaflumizone resistance were observed. These results imply that once resistance to metaflumizone has evolved in the field, its application should be suspended immediately and it should be substituted with chemicals that do not show cross-resistance, such as abamectin, beta-cypermethrin, chlorantraniliprole, or other insecticides. 

## Figures and Tables

**Table 1:** Toxicity of metaflumizone with and without TPP/DEM/PBO to susceptible and Meta-SEL populations of *P. xylostella.*

Strain	Synergist	LC50(mg/L) 95%CL	Slope ± SE	SR ^a^	N ^b^
SS	None	1.47 (1.18~1.85)	2.23 ± 0.27	-	240
	TPP	1.44 (0.99~2.60)	1.45 ± 0.23	1.02	193
	DEM	0.93 (0.63~1.33)	1.49 ± 0.25	1.58	211
	PBO	1.36 (0.99~1.95)	1.47 ± 0.22	1.08	185
Meta-SEL (G27)	None	1968.31 (1544.16~2507.72)	1.82 ± 0.25	-	232
	TPP	1055.82 (696.59~1406.49)	2.05 ± 0.34	1.86	186
	DEM	1387.59 (987.70~1827.14)	2.22 ± 0.38	1.42	179
	PBO	1585.70 (1098.49~2205.19)	1.73 ± 0.32	1.24	177

^a^ Synergism ratio was calculated as LC_50_ of metaflumizone/metaflumizone+TPP or EDM or PBO. ^b^ Number of larvae tested, excluding controls.

**Table 2:** Cross-resistance of resistant strain of diamondback moth to other insecticides.

Insecticide	Strain	LC50 (95% CL) mg·L^−1^	Slope ± SE	χ2	df	*p*	RR
indoxacarb	SS	1.69 (1.13~3.44)	1.56 ± 0.38	0.42	2	0.81	11.63
	G23	19.66 (13.65~34.01)	1.54 ± 0.32	3.98	3	0.26	
spinosad	SS	0.55 (0.37~1.03)	1.51 ± 0.27	2.23	4	0.69	1.75
	G23	0.95 (0.65~1.52)	1.07 ± 0.16	3.98	5	0.55	
spinetoram	SS	0.08 (0.03~0.14)	1.33 ± 0.32	0.81	3	0.85	3.52
	G23	0.20 (0.15~0.28)	1.64 ± 0.22	4.41	5	0.49	
abamectin	SS	0.07 (0.05~0.08)	1.83 ± 0.21	2.50	4	0.65	2.81
	G23	0.18 (0.08~0.29)	1.44 ± 0.30	2.12	2	0.35	
beta-cypermethrin	SS	6.51 (4.35~16.71)	1.71 ± 0.48	2.82	2	0.24	0.71
	G23	4.67 (3.01~10.15)	1.18 ± 0.28	0.33	3	0.95	
chlorfenapyr	SS	0.41 (0.29~0.81)	1.92 ± 0.40	2.49	2	0.29	0.49
	G22	0.32 (0.16~0.57)	0.81 ± 0.20	1.16	4	0.88	
diafenthiuron	SS	21.44 (16.45~28.96)	2.09 ± 0.31	2.89	4	0.58	0.79
	G23	16.96 (11.64~24.48)	1.30 ± 0.21	1.51	4	0.83	
chlorantraniliprole	SS	0.07 (0.03~0.11)	1.27 ± 0.23	0.17	3	0.98	2.16
	G23	0.15 (0.08~0.22)	1.39 ± 0.24	5.07	4	0.28	
BT (WG-001)	SS	0.89 (0.49~5.17)	1.43 ± 0.41	0.05	3	1.00	3.34
	G22	2.98 (1.49~12.60)	0.89 ± 0.19	3.51	5	0.62	
chlorfluazuron	SS	1.34 (0.86~1.94)	1.39 ± 0.24	2.24	4	0.69	0.97
	G22	1.29 (0.95~1.91)	1.71 ± 0.32	1.58	3	0.66	

**Table 3:** Stability of resistance to metaflumizone in UNSEL population of *P. xylostella* when reared without insecticidal exposure.

G ^a^	N	LC50 (mg/L) (95%CL)	Slope ± SE	χ2	*p*	df	RR ^b^	DR ^c^
G28	210	1599.13 (1289.07~1983.45)	2.14 ± 0.27	1.58	0.81	4	1087.85	-
G31	210	668.42 (420.28~1355.04)	0.97 ± 0.20	1.12	0.89	4	454.71	58.20
G32	179	795.37 (475.42~2600.78)	1.36 ± 0.33	0.49	0.92	3	541.07	50.26
G34	187	329.15 (234.62~509.16)	1.68 ± 0.33	0.98	0.81	3	223.91	79.42
G35	214	127.07 (76.55~255.38)	1.03 ± 0.24	1.85	0.76	4	86.44	92.05
G36	209	33.81 (20.57~51.23)	1.08 ± 0.20	4.00	0.41	4	23.00	97.89
G39	219	1.82 (0.46~3.17)	1.04 ± 0.28	0.91	0.82	3	1.23	99.89
G40	183	3.71 (2.34~11.10)	1.84 ± 0.45	1.63	0.65	3	2.53	99.77

^a^ Generation of selection with metaflumizone. ^b^ Resistance ratio = LC_50_ of the UNSEL population / LC_50_ of the SS population. ^c^ Depression rate = (RR_G_ − RR_G_ + 1) / RR_G_.
